# Screening for Depression and Anxiety in People With Diabetes and a Foot Ulcer in the Podiatry Clinic

**DOI:** 10.1002/jfa2.70208

**Published:** 2026-09-17

**Authors:** Emma Fletcher, Matthew Allen, Andrew Sharpe, Muneeb Ibad, Mathilde Mordaunt, Rosie Callen, Andy Gorman, Heather Schofield, Edward Jude, Adam Robinson, Reena Patel, Sheena Thayyil, Hermione Price, Edward Gee, Boris Mankovsky, George Dunn, Michael Edmonds, Neil Reeves, Adrian Heald

**Affiliations:** ^1^ Diabetes and Endocrinology Salford Royal Hospital Salford UK; ^2^ Department of Podiatry Salford Royal Hospital Salford UK; ^3^ University of Manchester Manchester UK; ^4^ Tameside and Glossop Integrated Care NHS Foundation Trust Ashton‐under‐Lyne UK; ^5^ Spinney Hill Medical Centre Leicester UK; ^6^ Diabetes Centre Leicester Royal Infirmary Leicester UK; ^7^ University of Southampton Southampton UK; ^8^ Institute of Gerontology Kyiv Ukraine; ^9^ East Cheshire Trust Macclesfield UK; ^10^ Kings College Hospital London UK; ^11^ Medical School University of Lancaster Lancaster UK

**Keywords:** anxiety, depression, diabetes, diabetes distress, foot ulcer, polypharmacy

## Abstract

**Introduction:**

There is close association between experience of diabetes‐tissue complications and depression/psychological distress. Our aim was to describe the results of prospective screening for anxiety/depression in a high‐risk multidisciplinary foot hospital clinic where people present with diabetes foot ulceration.

**Methods:**

Fifty patients attending our podiatry clinic participated in this study. All had active foot ulceration. The scales completed were GAD‐7 for anxiety/PHQ‐9 for depression plus 2‐specific diabetes distress scale items (16 + 2 questions). Spearman's rho(ρ) was utilised for correlational analysis due to the skewed distributions of variables. Medical history was obtained from primary and secondary care electronic health records. Inclusion criteria were type 1 or 2 diabetes and active foot ulceration.

**Results:**

Eight patients were diagnosed with type 1 diabetes and 42 with type 2 diabetes. Mean (standard deviation) (sd) age of the study population was 65.4 (12.9) years (*n* = 50) of which 86.7% were men. Median time since diagnosis was 14.5 (3.1) years. 42% of the patients had CKD (estimated GFR < 60 mL/min/1.73 m^2^). Median (IQR) for PHQ‐9 was 9 (3–16) and GAD‐7 was 4 (0.5–9.0) (mild‐moderate median scores for both). The median (IQR) of number of comorbidities was 8.3 (3.9); number of separate drugs was 10.5 (4.1). Depression/anxiety scores correlated highly positively with each other (Spearman's rho 0.791, *p* < 0.001) and with the diabetes distress score. Age was significantly and inversely correlated with anxiety (rho −0.472, *p* = 0.017) and depression (rho −0.250, *p* = 0.040). The PHQ‐9 questionnaire score for depression was higher in people taking more individual pharmacological agents (0.490, *p* = 0.001) and in people with a higher HbA1c (rho 0.421, *p* = 0.007) and correlated with the total of the 2 diabetes distress score items (rho 0.456, *p* = 0.006). Ulcer healed status at 12 weeks was not associated with any questionnaire score. Insulin treatment was associated with higher PHQ‐9 score.

**Conclusion:**

In this prospective study, we found a high correlation between depression/anxiety symptoms and diabetes foot ulceration, both recognised and unrecognised (until assessment). Both depression and anxiety symptoms declined with age. Higher depression scores associated with a higher current HbA1c and with greater number of prescribed medications.

## 1 Introduction

There is an established close association between the manifestation of diabetes‐tissue complications and either preceding or concurrent depression/psychological distress [1, 2].

In spite of advances in the detection and treatment of diabetes‐related foot problems, foot ulceration remains an important cause of disability for people with diabetes [3]. Up to a quarter of all people with diabetes develop a diabetes foot ulcer (DFU) at some point during their lives [4]. These ulcers are difficult to heal, are frequently recurrent, often leading to amputation and are associated with a high mortality rate [5, 6].

There is strong evidence linking diabetes foot ulceration with various psychosocial challenges, particularly stress, anxiety, fear and depression [7, 8, 9]. These psychological factors are not only common in patients with DFUs but are also believed to influence wound healing processes directly [10].

Comorbid depression is strongly associated with adverse health outcomes in people with diabetes including poor glycaemic control, increased risk of complications, higher mortality rate, decreased concordance with medications decreased concordance with dietary recommendations and increased healthcare costs [1].

Furthermore, higher diabetes distress (DDSS) and higher depressive symptoms (PHQ‐9 scale) have been linked with female sex, younger age, fewer years after diagnosis and obesity [11].

A previous meta‐analysis reported that nearly half of patients with diabetes related foot ulcers had clinically significant symptoms of depression [12].

Our aim was to quantify the degree of anxiety and depression in patients who had presented and were being treated for diabetes related foot ulceration in a specialist podiatry clinic.

## 2 Methods

Consecutive individuals attending our high risk podiatry clinic with active foot ulceration participated in this study. Eight patients were diagnosed with type 1 diabetes and 42 with type 2 diabetes.

The scales completed were GAD‐7 for anxiety (scale range 0–21) [13]/PHQ‐9 (scale range 0–27) for depression [14]/plus 2‐specific diabetes distress scale items [15] (16 + 2 questions). The 2 diabetes distress score items were 1 > I am feeling overwhelmed by the demands of living with diabetes? 2 > I am feeling that you are often failing with my diabetes regimen addressed specific areas not covered by the GAD‐7 or PHQ‐9 scales.

Medical history was obtained from primary and secondary care electronic health records.

This study was conducted within a Multidisciplinary Diabetic Foot Clinic (MDFC) over 12 months (2025–2026). Patients attending the clinic were approached whilst seated in a clinical cubicle following completion of their consultation with the multidisciplinary team. Verbal informed consent was obtained after the researcher introduced the study and provided a clear explanation of its purpose. Reassurance was given regarding confidentiality, the voluntary nature of participation, aftercare and established referral pathways.

Ethics statement: This initiative was approved as a service improvement project by the Northern Care Alliance NHS Foundation Trust (Reference 2025: NCA 12.2).

Data were collected using paper‐based questionnaires, which participants could complete independently or with assistance. Assistance was commonly requested due to visual impairment, low literacy levels or personal preference, ensuring accessibility and inclusivity. Upon completion, questionnaires were scored in real time, with results reviewed to identify levels of psychological distress, particularly elevated scores on the PHQ‐9 and GAD‐7 scales.

Participants with scores indicating a high level of anxiety and/or depression were assessed for immediate clinical urgency and offered referral to clinical psychology services and their general practitioner (GP). A follow‐up letter was sent to the patient's GP to communicate elevated scores and recommend further mental health assessment. Participants with lower scores were provided with written supported self‐management resources entitled ‘Living Well with a Long‐Term Health Condition [16]. This resource comprised IMPARTS booklets addressing key aspects of maintaining an active and healthy lifestyle whilst living with a long‐term condition. Participants were invited either to review the material during the consultation or to take it home for ongoing use.

### 2.1 Statistics

Descriptives are presented as means with standard deviations and medians with interquartile ranges for normally distributed and skewed variables, respectively. Spearman's rho(*ρ*) was utilised for correlational analysis and Mann–Whitney *U* test for nonparametric comparisons of psychological factors by predictor group.

## 3 Results

Sixty people were approached for the study. One woman and nine men declined to complete the questionnaire, leaving 50 active participants.

Mean (standard deviation) (sd) age of the study population was 65.4 (12.9) years (*n* = 50) of which 86.7% were men (Table 1). Forty‐two percent of the patients had CKD (estimated GFR of less than 60 mL/min/1.73 m^2^).

**TABLE 1:** Descriptives.

	Mean ± SD/median (IQR)/frequency (%)
Age	65.4 ± 12.9 years
Sex (male)	39/50 (78%)
Time since diagnosis (years)	14.5 ± 3.1
DDS	3 (2–7)
PHQ‐9	9 (3–16)
GAD‐7	4 (0.5–9)
No of comorbidities	8.3 ± 3.9
No of drugs	10.5 ± 4.1
Amputation (major and minor)	14 (31.1%)
Recurrent ulcers	15 (45.5%)
Number healed	17 (48.5%)
eGFR (mL/min/1.73 m^2^)	57.7 ± 27.2
CKD (eGFR < 60 mL/min/1.73 m^2^)	21 (42%)
HbA1c (mmol/mol and %) (median)	55.6 (7.2)

Median time since diagnosis was 14.5 (3.1) years. The median (IQR) for PHQ‐9 was 9 [3, 4, 5, 6, 7, 8, 9, 10, 11, 12, 13, 14, 15, 16] and GAD‐7 was 4 (0.5–9.0) (mild‐moderate median scores for both). The median (IQR) of number of comorbidities was 8.3 (3.9); drugs was 10.5 (4.1). 32/50 (64%) patients were treated with insulin.

**FIGURE 1:**
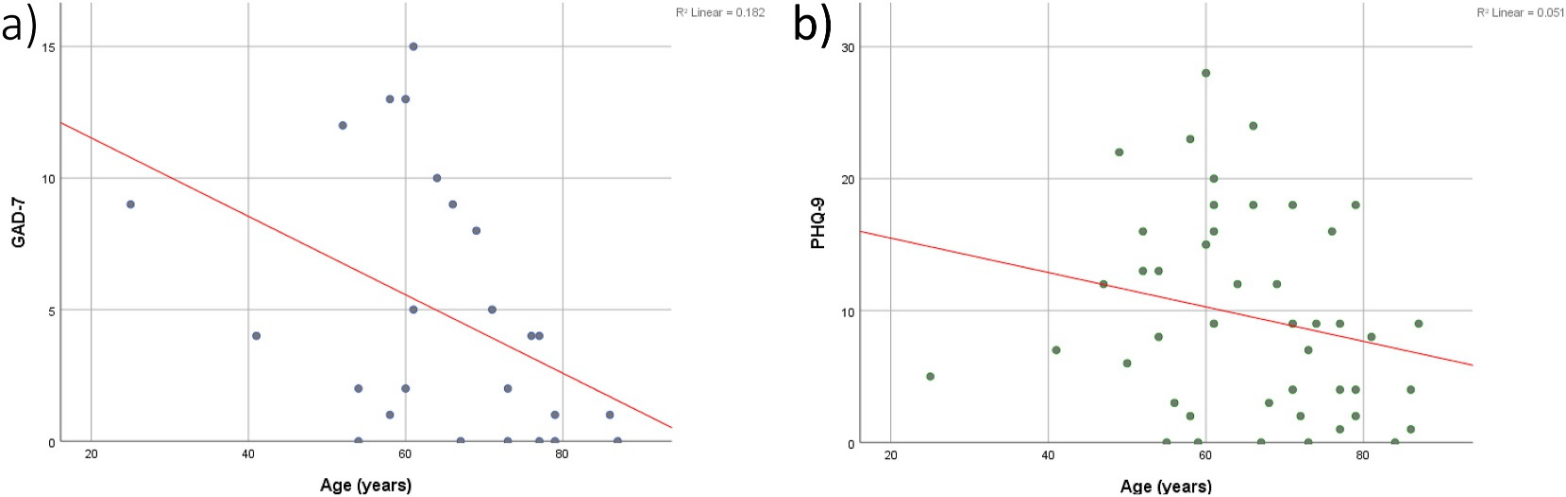
(a) GAD‐7 score versus age (years). (b) PHQ‐9 score versus age (years).

Depression and anxiety scores correlated highly positively with each other and with the diabetes distress score (0.791, *p* < 0.001) (Table 2). Age was significantly and inversely correlated with anxiety (Spearman's rho −0.472, *p* = 0.017) and depression (rho −0.250, *p* = 0.040) (Figure 1a,b), with younger adults reporting higher levels of anxiety and depression. The median (IQR) for PHQ‐9 (scale range 0–27) was 9 [3, 4, 5, 6, 7, 8, 9, 10, 11, 12, 13, 14, 15, 16] and GAD‐7 (scale range 0–21) was 4 (0.5–9.0) (mild‐moderate median scores for both).

**TABLE 2:** Correlations (Spearman's rho).

Variable	Measure	DDS	PHQ9	GAD7	Age	Comorbidities	No of drugs	eGFR	Ulcer area	HbA1c
DDS	Correlation coefficient	1.000	0.456**	0.512*	−0.406*	0.102	0.292	−0.105	−0.187	0.389*
	Sig. (2‐Tailed)		0.006	0.018	0.014	0.577	0.099	0.562	0.290	0.023
	*N*		35	21	36	32	33	33	33	34
PHQ9	Correlation coefficient		1.000	0.791**	−0.250*	0.292	0.490**	0.005	−0.167	0.421**
	Sig. (2‐Tailed)			0.000	0.040	0.071	0.001	0.974	0.310	0.007
	*N*			24	45	39	41	40	39	40
GAD7	Correlation coefficient			1.000	−0.472*	0.189	0.150	−0.022	0.504	0.143
	Sig. (2‐Tailed)				0.017	0.426	0.516	0.926	0.800	0.546
	*N*				25	20	21	20	23	20
Age	Correlation coefficient				1.000	0.101	−0.096	0.066	−0.165	−0.287
	Sig. (2‐Tailed)					0.537	0.546	0.680	0.31	0.069
	*N*					40	42	41	40	41
Comorbidities	Correlation coefficient					1.000	0.580**	−0.270	−0.175	0.402*
	Sig. (2‐Tailed)						< 0.001	0.092	0.315	0.011
	*N*						40	40	35	39
No of drugs	Correlation coefficient						1.000	−0.83	−0.413	0.419**
	Sig. (2‐Tailed)							0.252	,011	0.007
	*N*							41	37	40
eGFR	Correlation coefficient							1.000	−0.068	0.026
	Sig. (2‐Tailed)								0.732	0.875
	*N*								28	40
Ulcer area	Correlation coefficient								1.000	−0.081
	Sig. (2‐Tailed)									0.639
	*N*									36
HbA1c	Correlation coefficient									1.000
	Sig. (2‐Tailed)									
	*N*									

Ulcer maximum diameter at presentation varied between 3 and 38 mm. 24/50 individuals showed evidence of active bacterial infection on the basis of bacterial swab taken at presentation with antimicrobial therapy initiated as indicated. No Charcot cases were included.

The PHQ‐9 questionnaire score for depression was higher in people taking more individual pharmacological agents (rho 0.490, *p* = 0.040) and in people with a higher HbA1c (rho 0.421, *p* = 0.007) as for the total of the 2 diabetes distress score items (rho 0.456, *p* = 0.006) focusing on feeling overwhelmed by the demands of living with diabetes and the feeling of failing with the agreed diabetes regimen. The median (IQR) of number of comorbidities was 8.3 (standard deviation 3.9); and median number of separate drugs was 10.5 (standard deviation 4.1).

Questionnaire scores were unrelated to time to foot ulcer area at presentation, chronic kidney disease status (eGFR) of number of comorbidities. Ulcer healed status at 12 weeks was not associated with any questionnaire score (Table 3). Insulin treatment was associated with a higher PHQ‐9 score at the time of interview (Table 3).

**TABLE 3:** Comparisons (Mann–Whitney U).

	Significance *p* and (number)		
	DDS	PHQ‐9	GAD‐7
Sex	1.0 (35)	0.08	0.72
Previous amputation	0.24 (35)	0.26	0.6
Recurrent ulceration	0.19 (35)	0.211	0.37
CKD status	0.80 (33)	0.48	0.51
Ulcer healed at 12 weeks post presentation	0.67 (26)	0.053	0.097
Use of insulin	0.14 (30)	0.008	0.37
		Mean rank—Yes—21.5	
		Mean rank—No—12.6	
GLP1 analogues	0.76 (32)	0.27	0.84
HbA1c = > 58	0.107 (34)	0.012	0.44
		Mean rank (≥ 58)—26.5	
		Mean rank (< 58)—16.9	

## 4 Discussion

We found a high correlation between depression and anxiety symptoms and diabetes foot ulceration, both recognised and unrecognised (until the assessment). Both depression and anxiety symptoms declined with age. Higher depression scores associated with a higher current HbA1c and with a greater number of prescribed medications.

Our study findings strongly accord with an earlier study by Pearson et al. [17], which found a high prevalence of depressive symptoms both recognised and unrecognised in people with diabetes and foot ulcers. Depressive symptoms were associated with overall poorer diabetes self‐management and health‐related quality of life (HRQoL). As we found, there was no association in the Pearson study between depressive symptoms and ulcer outcomes at six months follow‐up. The authors also reported that the prolonged use of antidepressants appeared to be ineffective in the majority of cases.

We report a negative association with increasing age for both PHQ‐9 and GAD‐7. This accords with a number of studies looking at psychological health in people with diabetes [2, 9, 11, 12].

We found a strong positive association between the 2 diabetes distress score items totalled score and both PHQ‐9 total and GAD‐7 total scores. The diabetes distress score items focussed on feeling overwhelmed by the demands of living with diabetes and the feeling of failing with the agreed diabetes regimen.

HbA1c had a positive association with the PHQ‐9 score. A previous study [18] did not find such an association, but this study was not conducted in people with active diabetes foot ulceration. Ours may be the first report of this association in this specific context, whereas PHQ‐9, although not relating to comorbidity number, did relate to the number of medications prescribed as previously reported in a study using the UK Clinical Practice Research Datalink (CPRD) (years 2000–2018) [19]. Two fifths of the patients had CKD. This is in accordance with the high burden of comorbidity and shortened life expectancy seen in people with diabetes foot complications [6].

Use of insulin was associated with higher PHQ‐9 scores. Previous studies have indicated that insulin use can lead to higher levels of depression and anxiety. Specifically, a 2018 metanalysis looking mainly at cross‐sectional studies reported that insulin therapy was significantly associated with the risk of depressive symptoms occurring [20].

A recent scoping review reported that psychological distress impairs DFU healing [10]. We did not see any relation between questionnaire scores and healed status at 12 weeks post presentation. However, we were not directly measuring stress here. Chronic stress has been found to extend inflammation and weaken immune function [10], whereas anxiety and depression have been associated with reduced self‐care and directly impaired wound healing, further hindering recovery [8, 10]. This highlights the significant impact of psychological factors on the DFU healing process.

The management of diabetic foot ulcers, including wound care strategies, decisions regarding lower‐limb amputation and postoperative care, has been shown to influence patients' health‐related quality of life and interpersonal relationships. Evidence indicates that individuals with diabetic foot complications experience substantial psychosocial burden, including reduced mobility, social isolation and impaired role functioning [21]. Additionally, the socioeconomic impact, such as loss of employment, reduced income and increased dependency on caregivers, further contributes to diminished well‐being [22, 23]. Caregivers themselves may also experience psychological strain and altered social dynamics.

Accordingly, optimal management should not be limited to pharmacological treatment and clinical parameters but should adopt a multidisciplinary approach that incorporates psychosocial and socioeconomic considerations [24]. These factors are well‐documented contributors to adverse mental health outcomes, including increased prevalence of depression and psychological stress among patients with diabetic foot disease.

A factor that should be taken into account in any interpretation of our findings is that socioeconomic disadvantage predisposes both to depression and to cardiometabolic disease at a population level [25] as well as to foot ulceration itself [26].

As this was a cross‐sectional study involving a one‐off assessment, it is not possible to say whether the depression or anxiety preceded the foot ulcer or was a consequence of it. However, it is well established that high levels of glucose do predispose, through various mechanisms, to the development of diabetes foot ulcers and that depression and diabetes distress, do associate with less good glycaemic control and reduced self‐care behaviours as evidenced in a recent meta‐analysis [27].

Regarding limitations, we accept that there is no comparator group in our study in relation to people with no history of diabetes foot ulceration. Also, we did not have sufficient numbers to differentiate type 1 from type 2 diabetes individuals and we were not able to define an age threshold for more prevalent affective symptoms. However, the point of the study was simply to describe the prevalence and degree of depression and anxiety in a high risk foot clinic. Existing research on psychological and emotional aspects of diabetes foot ulceration is diverse, using a wide variety of study designs, concepts, and tools [24]. Most studies focus on quantitative psychological factors. There is a clear need for further exploration of other relevant aspects to enhance patient support.

In summary, we found that we have found a high correlation between depressive symptoms and diabetes foot ulceration, albeit both recognised and unrecognised (until assessment). Both depression and anxiety symptoms declined with age, suggesting that particular attention in relation to screening be focussed on younger people with foot ulceration. Higher depression scores associated with a higher current HbA1c and with a medication burden.

We report that depression and anxiety scores were lower in older individuals with a foot ulcer. This suggests that psychological interventions in this group may need to address more general issues than specific diabetes related challenges.

Consideration should be given to the concept of “diabetes distress” or “diabetes burnout,” particularly among individuals with type 1 diabetes or those requiring intensive insulin therapy for the treatment to type 2 diabetes. This phenomenon is characterised by emotional exhaustion, reduced motivation for self‐management and feelings of being overwhelmed [11], related to the chronic demands of diabetes care, and has been associated with poorer psychological outcomes and suboptimal glycaemic control [28, 29, 30].

Psychological distress, particularly stress, anxiety and depression, may impair the healing of diabetes foot ulcers [10]. The findings highlight the potential importance of addressing psychological factors as a core component of diabetes foot ulcer management.

Further work should consider proposing practical strategies to address these findings. In particular, it would be pertinent to evaluate whether multidisciplinary diabetic foot clinics incorporate dedicated psychological support, such as access to a psychiatrist, psychologist or structured therapeutic interventions, as part of routine care for patients with nonhealing diabetic foot ulcers and those undergoing pre and postamputation management. This is especially relevant in the context of limited access to mental health services within primary care in the United Kingdom National Health Service and other healthcare systems across the world.

## Author Contributions

All authors made relevant and substantial contributions to this paper.

## Funding

The authors have nothing to report.

## Ethics Statement

This initiative was approved as a service improvement project by the Northern Care Alliance NHS Foundation Trust (Reference 2025: NCA 12.2).

## Consent

All participants gave written permission to be included in this study.

## Conflicts of Interest

The authors declare no conflicts of interest.

## Data Availability

The data that supports the findings of the study are available on reasonable request.
